# Pyramidal core-shell quantum dot under applied electric and magnetic fields

**DOI:** 10.1038/s41598-020-65442-x

**Published:** 2020-06-02

**Authors:** J. A. Osorio, D. Caicedo-Paredes, J. A. Vinasco, A. L. Morales, A. Radu, R. L. Restrepo, J. C. Martínez-Orozco, A. Tiutiunnyk, D. Laroze, Nguyen N. Hieu, Huynh V. Phuc, M. E. Mora-Ramos, C. A. Duque

**Affiliations:** 1 Grupo de Materia Condensada-UdeA, Instituto de Física, Facultad de Ciencias Exactas y Naturales, Universidad de Antioquia UdeA, Calle 70 No. 52-21, Medellín, Colombia; 2 Facultad de Ingeniería, Universidad de Antioquia UdeA, Calle 70 No. 52-21, Medellín, Colombia; 3 Department of Physics, “Politehnica” University of Bucharest, 313 Splaiul Independenței, Bucharest, RO-060042 Romania; 4 Universidad EIA, CP 055428 Envigado, Colombia; 5 Unidad Académica de Física, Universidad Autónoma de Zacatecas, Calzada Solidaridad esquina con Paseo la Bufa S/N, C.P, 98060 Zacatecas, Zac. México; 6 Instituto de Alta Investigación, CEDENNA, Universidad de Tarapacá, Casilla 7D, Arica, Chile; 7 Institute of Research and Development, Duy Tan University, Da Nang, 550000 Viet Nam; 8 Division of Theoretical Physics, Dong Thap University, Cao Lanh, 870000 Viet Nam; 9 Centro de Investigación en Ciencias-IICBA, Universidad Autónoma del Estado de Morelos, Av. Universidad 1001, CP 62209 Cuernavaca, Morelos Mexico

**Keywords:** Materials science, Nanoscience and technology, Physics

## Abstract

We have theoretically investigated the electronic states in a core/shell pyramidal quantum dot with GaAs core embedded in AlGaAs matrix. This system has a quite similar recent experimental realization through a cone/shell structure [Phys. Status Solidi-RRL 13, 1800245 (2018)]. The research has been performed within the effective mass approximation taking into account position-dependent effective masses and the presence of external electric and magnetic fields. For the numerical solution of the resulting three-dimensional partial differential equation we have used a finite element method. A detailed study of the conduction band states wave functions and their associated energy levels is presented, with the analysis of the effect of the geometry and the external probes. The calculation of the non-permanent electric polarization via the off-diagonal intraband dipole moment matrix elements allows to consider the related optical response by evaluating the coefficients of light absorption and relative refractive index changes, under different applied magnetic field configurations.

## Introduction

Since the 1970s to date, the interest in the development and understanding of the physical bases that underline the operation of novel electronic and optoelectronic components has been growing. These state-of-the-art systems allow to improve the operating capabilities of sensors, switches, emitters, filters, and optical and electronic transport systems, among others. Applications in the construction of high resolution images in biomedicine, solid state lasers, and for photovoltaic generation in solar panels are known. Along these lines, the increasing use of low-dimensional semiconductor nanostructures has become a key element for the development of the most advanced devices. Among the more notorious we can cite: the two-dimensional quantum wells (QW), the one-dimensional quantum well-wires (QWW), and the zero-dimensional quantum dots (QD). The latter are widely referred in the literature as artificial atoms, due to the discrete nature of the electronic structure for the confined carriers. Several methods have been developed for obtaining the zero-dimensional QDs: Molecular Beam Epitaxy (MBE), Chemical Vapour Deposition (CVD), and Stranski Krastanov growth. This last technique has been implemented with highly reproducible results to obtain self-assembled pyramidal QDs in systems where the zero-dimensional system has been formed by the coupling between two materials with sufficiently different lattices constants to give rise to strain effects, which is finally the basis for the formation of QD. Chemical synthesis is another technique, widely employed to produce, in this case, core-shell QDs and nanoparticles.

Theoretical studies of the electronic and optical properties of carriers confined within QDs have included a wide variety of shapes and external effects on the system. There are reports of spherical, cubic, pyramidal, conical, core-shell, ellipsoidal, and truncated pyramidal QDs. The studies have considered effects of stationary electric and magnetic fields, hydrostatic pressure, temperature, non-resonant intense laser radiation, optical phonons, stresses on the surfaces, wetting layer (WL), presence of shallow impurities, and excitonic correlations, among others.

Using an analytical solution -valid for structures with apical angle less than *π*/6-, and contrasting and confirming the results with those obtained by means of numerical solutions using a finite element method (FEM), Bahramiyan *et al*., Gil *et al*., and Niculescu *et al*.^1–5^ have studied the electronic structure for electrons confined in pyramidal and conical QDs. They report, among others, the symmetry properties of wave functions, shallow donor impurity effects, axially applied magnetic field effects, effects of elliptically polarized radiation, the second and third harmonics generation, and the light absorption and relative changes of refractive index coefficients for allowed intraband transitions. Bahramiyan *et al*., have extended their study to include the shallow donor impurities and WL effects but using a finite difference method to obtain the electronic structure^6^. A GaAs pyramidal structure, with square base, has also been studied by Califano *et al*.^7^ to validate the comparison between theory and experiment for excitonic transitions. They performed a 3D-diagonalization method with the product of sine-functions as basis, and took into account the spatial dependence of the effective mass of both electrons and holes. Through FEM, the effects of strain, WL, optical phonons, and non-parabolicity of the conduction band have been included in the study of the electronic structure for InAs/GaAs pyramidal QDs with square base and it was found that: (*i*) the hydrostatic strain is mostly confined in the QD region and (*ii*) the anisotropic strain goes from the QD towards the barrier material. In the case of vertically coupled pyramids, the authors found that a splitting of the electronic levels appears as the separation between the pyramids decreases^8,9^. Also, several shapes of InAs/GaAs 3D-heterostructures (cuboid, cylindrical, pyramidal, conical, and lens-shaped) have been employed to carry out a comparative study of the energy structure for electrons and holes without electrostatic correlation. The authors, in particular, report the properties of the ground and first excited states considering different QD dimensions controlled through the aspect ratio variation.

The piezoelectric and strain effects, combined with lateral electric fields and the presence of a WL, have been implemented in the study of the absorption coefficient (linear and third order corrections) and the photoluminescence peak energy transition (relative to neutral and charged excitons) in InGaAs/GaAs, InGaN/GaN, and CdTe/ZnTe QD with pyramidal and conical lens geometries^10–12^. The calculations have been made using combinations of the FEM numerical techniques to obtain the energies and wave functions of a single particle^10^, while in the case of the single-particle electron and hole states under the lateral electric field the authors made use of the 8-band $\overrightarrow{k}\cdot \overrightarrow{p}$ theory^10–12^.

A systematic study related to the WL effects on the electronic structure of InAs/GaAs pyramidal quantum dots has been reported in the works by Sabaeian *et al*.^13–15^. The authors have reported the optical absorption and relative changes in the refractive index coefficients as a function of an applied magnetic field. Their main findings can be summarized as follows: (*i*) the *P*-like → *S*-like transition, which is an in-plane-polarized transition, is moderate and can be neglected and (*ii*) for the *WL* → *P*-like and *WL* → *S*-like transitions, which are in-plane and *z*-polarized transitions, respectively, the electronic as well as optical properties are strongly size-dependent. In the same line of research, and using the adiabatic approach, Hayrapetyan *et al*.^16^ have reported the absorption coefficient for electrons confined in conical QD with infinite confinement potential under the effects of an applied magnetic field. That work has allowed them to glimpse possible applications for the development of QD-LED. In this sense, it is worth noting that the adiabatic approach has demonstrated its immense value to reduce the computational cost in those problems where it is dealt with three-dimensional heterostructures with and without symmetries. In structures with azimuthal symmetry, for example, the adiabatic approach allows us to convert a 3D-problem into a 2D one, which finally, making use of the symmetry of the problem, leads to the numerical solution of a 1D-differential equation. Clearly, this approach is valid in those problems where the Hamiltonian can be separated into fast and slow movement components, as is the case of 3D-structures with much smaller height than the dimensions of the base or in 1D-problems such as QWW and quantum rings.

Core-shell heterostructures have been under intensive study in the last years due to their prospective applications in different scientific and technological areas^17^. The physical interest lies in the fact that by modulating the dimensions, the shapes, and their constituent materials, it is possible to perform the tuning and optimization of the optical properties, allowing their use in devices such as LEDs, detectors, lasers, and photovoltaic cells. A number of works have considered the electronic and optical properties of confined carriers in core-shell nanowires and QWWs^18–22^. By means of suitable combinations of the core and shell dimensions it is possible to modify the spatial location of the wave functions for the different confined electron states in a core-shell QD. Depending on the position of a donor impurity in the dot and by the relative change in the position of the wave functions, the electric dipole moment matrix elements for transitions between correlated and uncorrelated states associated with photoionization^23,24^, and for intra-band transitions processes involved with polaronic effects^25,26^ can be modified. The effects of temperature, hydrostatic pressure, and applied electric field on the electronic properties for electrons, impurities, and excitons confined in core/shell QDs have also been reported in the last five years^27–29^. Using the WKB method and the effective mass approximation, the energies of a confined electron in a GaAs/Ga_*x*_Al_1−*x*_ As core/shell QD were calculated assuming the presence of an applied electric field as a tuning parameter^30^. The results showed that the energy of the ground and first excited states are inverse functions of the applied electric field at a given dot radius. More complicated profiles of the conduction band structure have been considered for the core/shell QDs. For example, using the Hylleraas coordinates combined with a variational procedure, Chafai *et al*. have studied the excitonic spectrum in CdSe/ZnTe type II core/shell QDs^31^.

A fairly novel structure corresponding to a GaAs cone/shell submerged in AlGaAs matrix under vertically applied electric field has been first reported by Heyn *et al*.^32^. The experimental studies have included results of the photoluminescence spectrum revealing the presence of a discrete set of electronic levels and excitons and biexcitons states. The cone/shell shape of the structure was unveiled using the AFM technique. The simulations showed that without electric field effects the charge carriers (electron and hole) tend to be located in the apical region of the structure, giving rise to a spatially direct exciton and that through the applied electric field it is possible to polarize the system giving rise to a spatially indirect exciton with changes in lifetime ranging from nanoseconds up to milliseconds. The analysis of the probability distributions shows the evolution between QD and quantum ring induced by the electric field. To date there are no further known developments of this type of cone/shell novel structure nor about similar pyramidal/shell structures. Taking into account the high degree of development that pyramidal QDs have had, we consider that the implementation in the laboratory of a pyramid/shell QD to be viable without much effort. Therefore, using the work from Heyn *et al*.^32^ as a departing point, we have assumed the theoretical investigation of the pyramid/shell QDs as the subject of this research. We will go further and include the effects of a static magnetic field parallel to the vertically applied electric field. We shall focus our attention on the electronic structure, the wave function symmetries, and the intra-band optical absorption. The possible electric-field-induced appearance of indirect excitonic complexes, related to effective spatial separation of electron and hole states is briefly discussed as well. The article has the following organization: The theoretical framework is presented in section II. The section III contains the results and discussion. Finally, in the section IV we outline the conclusions.

## Theoretical Framework

**Figure 1:**
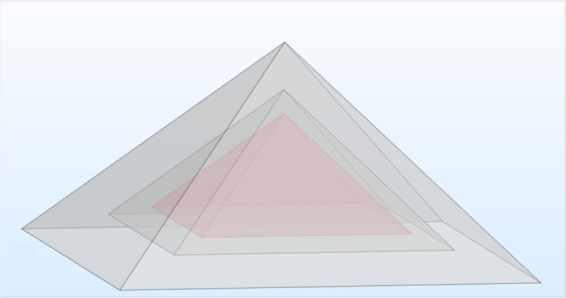
3D view of the pyramidal core-shell quantum dot.

**Figure 2:**
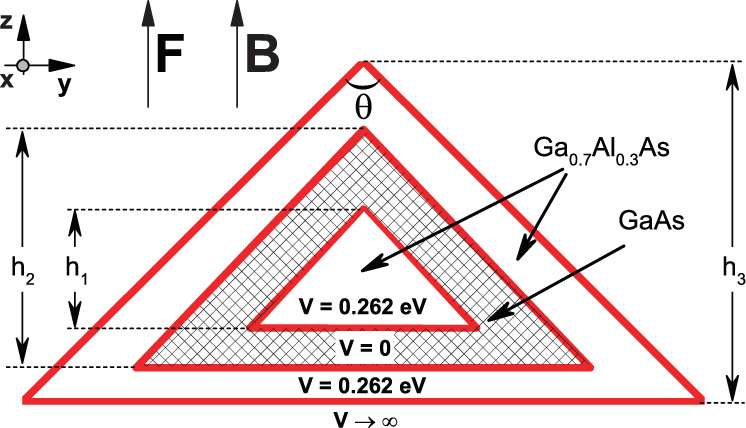
Front view of the pyramidal core-shell quantum dot (*yz* plane, *x* = *0*). *h*_*i*_ represents the height of each shell. *θ* is the vertex angle of the pyramid.

Figure 1 shows the 3D projection of the structure while a schematic view of the pyramidal core-shell quantum dot (PCSQD) is shown in Fig. 2, with *θ* labeling the vertex angle, and *h*_*i*_ the height of each pyramid. The center of gravity of the PCSQD is assumed to be at *z* = 0. So, our problem is to study the energy states and their corresponding wave functions for an electron confined in a pyramidal structure like the one shown in Fig. 1 and subjected to the effects of stationary electric or magnetic fields, both applied in the *z*-axis, parallel to the symmetric axis of the pyramid. Within the framework of the effective mass, the Hamiltonian for this problem, in Cartesian coordinates, takes the form:

1 $$\begin{array}{l}H=\frac{1}{2{m}_{w,b}^{\ast }}{(i\hslash \overrightarrow{\nabla }+e\overrightarrow{{\bf{A}}})}^{2}+V(x,y,z)+eFz,\end{array}$$

where *e* is the electron charge, ${m}_{w,b}^{\ast }$ is the effective mass (*b* means the barrier region or innermost and outermost pyramid and *w* means the well region or the pyramid in the center), and *V*(*x*, *y*, *z*) is the confinement potential for the PCSQD which is *V*_0_ in the innermost and outermost pyramid, zero for the pyramid in the center, and ∞ outside the PCSQD.

The particular gauge chosen to describe the magnetic field in the system implies the conditions

2 $$\begin{array}{c}\overrightarrow{{\rm{\nabla }}}\cdot \overrightarrow{{\bf{A}}}=0,\\ \overrightarrow{{\bf{A}}}=-\frac{1}{2}\overrightarrow{{\bf{r}}}\times \overrightarrow{{\bf{B}}},\end{array}$$

for the magnetic vector potential, where $\overrightarrow{{\bf{B}}}$ properly represents the field.

The expanded form of the Hamiltonian (Eq. (1)) gives

3 $$\begin{array}{ccc}H & = & -\frac{{\hslash }^{2}}{2{m}_{w,b}^{\ast }}{\overrightarrow{{\rm{\nabla }}}}^{2}+\frac{ie\hslash }{{m}_{w,b}^{\ast }}\overrightarrow{{\boldsymbol{A}}}\cdot \overrightarrow{{\rm{\nabla }}}+\frac{{e}^{2}}{2{m}_{w,b}^{\ast }}{\overrightarrow{{\boldsymbol{A}}}}^{2}\\  &  & +V(x,y,z)+eFz.\end{array}$$

Using the expression of the magnetic potential Eq. (2) the final form of the Hamiltonian Eq. (3) is:

4 $$\begin{array}{ccc}H & = & -\frac{{\hslash }^{2}}{2{m}_{w,b}^{\ast }}{\overrightarrow{{\rm{\nabla }}}}^{2}-\frac{ie\hslash }{{m}_{w,b}^{\ast }}\left(y,\frac{{\rm{\partial }}}{{\rm{\partial }}x},-,x,\frac{{\rm{\partial }}}{{\rm{\partial }}y}\right)\\  &  & +\frac{{e}^{2}{B}^{2}}{8{m}_{w,b}^{\ast }}({x}^{2}+{y}^{2})+V(x,y,z)+eFz.\end{array}$$

The energies and wavefunctions of the bound states can be obtained by solving the Schrödinger equation:

5 $$H{\Psi }_{i}(x,y,z)={E}_{i}{\Psi }_{i}(x,y,z\mathrm{)}.$$

The eigenvalues and eigenstates (Eq. (5)) are calculated with the software *COMSOL-Multiphysics*^33^, which uses a FEM to solve the partial differential equation numerically. A complete description of the *COMSOL-Multiphysics* licensed software that includes the foundation of the finite element method, the construction of meshes, the discretization of the differential equations, the methods to optimize the processes, the construction of geometries and the convergence criteria can be found in^34,35^. Since Ψ_*i*_(*x*, *y*, *z*) is finite, the Dirichlet boundary condition implies that any of its values outside the PCSQD are equal to zero, i.e. the wave functions are zero at the interfaces between the outermost pyramid and the infinite potential region (see Fig. 2). For layered structures such as the one in the current study, the Schrödinger equation interface accounts for the discontinuity in the effective mass by implementing the BenDaniel-Duke boundary conditions.

One of the optical coefficients to be evaluated in this work are the light absorption one, which derives from the imaginary part of the dielectric susceptibility. So, we have:

6 $$\alpha (\hslash \omega )=\left(\frac{\pi \omega }{c\,{n}_{r}{\epsilon }_{0}}\right)\frac{2}{V}\sum _{f\ne i}\,{|{\mu }_{\xi }^{f,i}|}^{2}\delta ({E}_{f}-{E}_{i}-\hslash \omega ),$$

and owing to take into account possible damping effects associated with intraband transitions induced by photon absorption, the Dirac delta term is usually substituted by a Lorentzian term, thanks to the well known relation

7 $$\delta ({E}_{f}-{E}_{i}-\hslash \omega )=\mathop{{\rm{l}}{\rm{i}}{\rm{m}}}\limits_{{\Gamma }_{fi}\to 0}\frac{1}{2\,\pi }\frac{{\Gamma }_{fi}}{{({E}_{f}-{E}_{i}-\hslash \omega )}^{2}+{\left(\frac{{\Gamma }_{fi}}{2}\right)}^{2}},$$

in which Γ_*fi*_ (=10 meV in this work) accounts for the corresponding damping rates. In the former expressions, *ω* represents the incident photon frequency, *c* is the speed of light in the vacuum, *n*_*r*_ is the static value of the refractive index, and ${\epsilon }_{0}$ is the vacuum permittivity. The quantities *E*_*f*_ and *E*_*i*_ are, respectively, the energy of the final state and the energy of the initial state of the light-induced intraband transition. Since we are assuming to work in the very low temperature case, the electron density per unit volume is taken to be 2/*V*, where *V* represents the PCSQD volume and the 2 indicates the possible spin contributions. This has to do with the situation in which a single electron would be excited towards the conduction band at low *T*﻿. In this work the electron density was taken as $3\times \backslash {10}^{22}{{\rm{m}}}^{-3}$. Finally, $\overrightarrow{\xi }$ is the unit vector representing the polarization of the -homogeneously intense- incident light (for instance, if the light is circularly polarized in the *xy*-plane, then $\overrightarrow{\xi }={\overrightarrow{e}}_{1}/\sqrt{2}\pm i{\overrightarrow{e}}_{2}/\sqrt{2}$, where ${\overrightarrow{e}}_{1}$ and ${\overrightarrow{e}}_{2}$ are the unit vectors along the *x*- and *y*-direction, respectively).

The general expression for the electric dipole moment matrix element, ${\mu }_{\xi }^{f,i}$, is the following:

8 $${\mu }_{\xi }^{f,i}=\langle {\Psi }_{f}|e\overrightarrow{\xi }\cdot \overrightarrow{r}|{\Psi }_{i}\rangle ,$$

in which *e* is the electron charge and $\overrightarrow{r}$ is the vector position.

In an analogous way, the expression for the coefficient of relative change of the refractive index comes from the real part of the dielectric susceptibility. Its final form reads:

9 $$\frac{\Delta n(\omega )}{{n}_{r}}=\frac{1}{2{\epsilon }_{0}{n}_{r}^{2}}\left(\frac{2}{V}\right)\sum _{f\ne i}\frac{|{\mu }_{\xi }^{f,i}{|}^{2}({E}_{f}-{E}_{i}-\hslash \omega )}{{({E}_{f}-{E}_{i}-\hslash \omega )}^{2}+{\left(\frac{{\Gamma }_{fi}}{2}\right)}^{2}}.$$

In Eqs. (6) and (9), the summation is carried out over all possible allowed inter-state transitions.

## Results and Discussion

As stated in the previous section, the present study makes use of a FEM to solve the eigenvalues differential equations. In particular, a self-adapting mesh has been used that includes tetrahedra in the volume region, triangles on the surfaces, edge elements at the intersection between two planes, and vertex elements at the intersection between three planes. For a pyramid with *h*_1_ = 5 nm, *h*_2_ = 20 nm, *h*_3_ = 30 nm, and *θ* = *π*/2, the used parameters are: 86365 tetrahedra, 11242 triangles, 564 edge elements, and 15 vertex elements, which guarantees a convergence of 0.1 meV for the fifteen lowest states that have been calculated. In this paper we report results for the thirteen lowest energy states.

**Figure 3:**
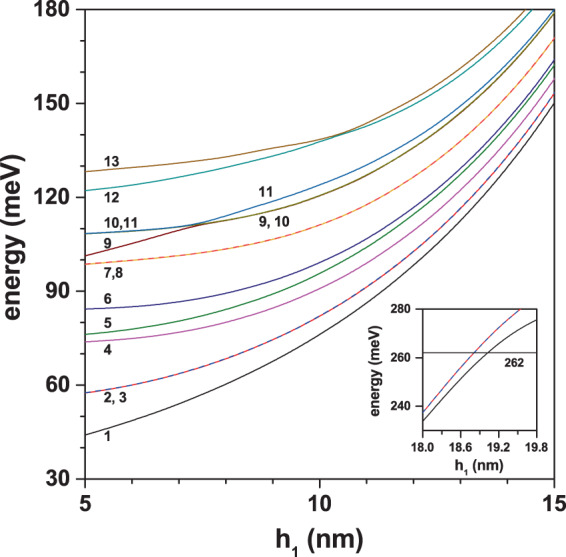
Energy of the lowest thirteen confined electron states in a GaAs–Ga_0.7_Al_0.3_ As pyramidal core-shell quantum dot as functions of the innermost height (*h*_1_). Calculations correspond to *F* = 0, *B* = 0, *θ* = *π*/2, *h*_2_ = 20 nm, and *h*_3_ = 30 nm. The inset shows the energies of the three lowest confined states when *h*_1_ approaches *h*_2_.

In Fig. 3 the energies of the thirteen lowest confined electron states in a GaAs–Ga_0.7_Al_0.3_ As PCSQD are depicted as functions of the innermost pyramid height. Calculations correspond to the situation in which the electric and magnetic field intensities are equal to zero, *θ* = *π*/2, *h*_2_ = 20 nm, and *h*_3_ = 30 nm. It is observed that all energy levels have a growing tendency as long as *h*_1_ augments. This is due to the progressive decrease in the volume of the GaAs layer where the electron is confined.

**Figure 4:**
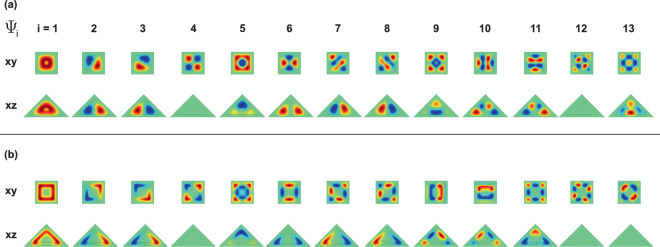
Pictorial view of the wave function projections (on *z* = 0 and *y* = 0 planes) for the thirteen lowest confined electron states in a GaAs–Ga_0.7_Al_0.3_ As pyramidal core-shell quantum dot. The depicted results correspond to *h*_1_ = 5 nm (**a**) and *h*_1_ = 15 nm (**b**) with *F* = 0, *B* = 0, *θ* = *π*/2, *h*_2_ = 20 nm, and *h*_3_ = 30 nm. In the scale of colors, the green indicates the zero value of the wave function whereas the red and blue are associated to the positive and negative maxima, respectively.

For *h*_1_ = 5 nm the levels (2, 3), (7, 8), and (10, 11) appear to be degenerate, while for *h*_1_ > 7.4 nm, the degenerate levels are (2, 3), (7, 8), and (9, 10). This degeneracy comes from the square symmetry of the base of the pyramid, with respect to an axis that passes through its center of gravity and the upper vertex (see Fig. 1). From Fig. 4 -where the projections of the wave functions of the first thirteen confined states onto the *xy*-plane (with *z* = 0) and onto the *xz*-plane (with *y* = 0) are shown-, it is possible to observe that, for example, the degenerate states Ψ_2_ and Ψ_3_ have *p*-like symmetry. The states Ψ_1_ and Ψ_5_ exhibit *s*-like symmetry and the state Ψ_4_ displays *d*-like symmetry. The states Ψ_2_ and Ψ_3_ appear rotated in the *xy*-plane due to an indeterminacy of the phase, which is typical to the used numerical method. By introducing a very small asymmetry in the dimensions of the base, clearly the Ψ_2_ and Ψ_3_ states would be oriented along the *x* and *y* perpendicular axes. In Fig. 4 the color scale is defined with green corresponding to zero, red to a maximum positive value, and the negative maximum for the blue. For Ψ_1_ in the *xy*-plane, with *h*_1_ = 5 nm, one notices that the wave function has finite values in the center of the structure (the wave function penetrates the center region), indicating the possibility to find the electron around the gravity center of the PCSQD, whereas for *h*_1_ = 15 nm, the electron will completely confine inside the GaAs layer. When comparing the behavior of Ψ_1_ in the *xz*-plane, it can be seen that for *h*_1_ = 5 nm the electron can be found both in the lateral regions and in the base of the GaAs pyramid, while for *h*_1_ = 15 nm, the probability density concentrates mainly towards the side walls of the central pyramid. Finally, note that the value zero of Ψ_4_ along the *xz*-plane, for both *h*_1_ = 5 nm and *h*_1_ = 15 nm, is consistent with the null value of the wave functions in the *xy*-plane along the *y* = 0 line. It is interesting to note from Fig. 4 that the Ψ_7_ and Ψ_8_ states have exactly the same symmetries as the Ψ_2_ and Ψ_3_ states and that, like the first two excited states, in the entire range of calculated *h*_1_-values, Ψ_7_ and Ψ_8_ are degenerate states. Here it should be noted that Ψ_7_ and Ψ_8_ double the number of Ψ_2_ and Ψ_3_ antinodes, which is in accordance with their higher energy values. At *h*_1_ = 7.35 nm an accidental degeneration appears with three states that have the same energy. From Fig. 4(a) it is observed that for *h*_1_ = 5 nm, the calculation of ${\Psi }_{10}^{2}+{\Psi }_{11}^{2}$ leads to a probability density very similar to that obtained with ${\Psi }_{9}^{2}$, while for *h*_1_ = 15 nm, in Fig. 4(b), the calculation of ${\Psi }_{9}^{2}+{\Psi }_{10}^{2}$ perfectly approximates ${\Psi }_{11}^{2}$. This is in agreement with the change of symmetries that is observed in *h*_1_ = 7.35 nm.

**Figure 5:**
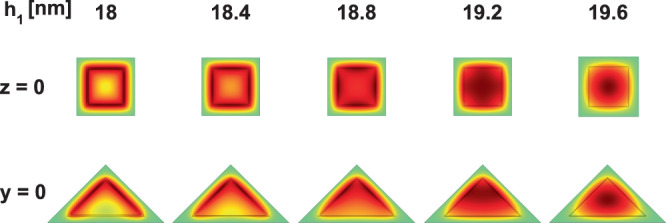
Pictorial view of the wave function projections (onto *z* = 0 and *y* = 0 planes) for the ground state in a GaAs–Ga_0.7_Al_0.3_ As pyramidal core-shell quantum dot, when *h*_1_ → *h*_2_. The setup of the structure is as in Fig. 4. The green color corresponds to a zero value whereas the red one is associated to the positive maxima.

Furthermore, the inset in Fig. 3 shows the evolution of the three lowest confined electron states in a PCSQD as *h*_1_ approaches *h*_2_ = 20 nm. That is, as the inner GaAs pyramid thickness approaches zero. Note that for *h*_1_ = 19 nm, the ground state energy reaches the value of the potential barrier (262 meV) and from there the wave functions overflow to the Ga_0.7_Al_0.3_ As region. This can be visualized in the fourth column of Fig. 5 where the, *z* = 0, *xy*-projections of the wave function for the ground state are presented as the thickness of the GaAs layer tends to zero. In that case, the electron is, actually, confined in a pyramid of Ga_0.7_Al_0.3_ As of height *h*_3_ with infinite external potential barriers. For 18.8 nm < *h*_1_ < 19 nm, only the ground state is confined within the GaAs region. Going from *h*_1_ = 18 nm towards *h*_1_ = 19.6 nm it is observed how the system evolves from a 2D-confinement in the GaAs region to a 3D-one in the Ga_0.7_Al_0.3_As structure.

**Figure 6:**
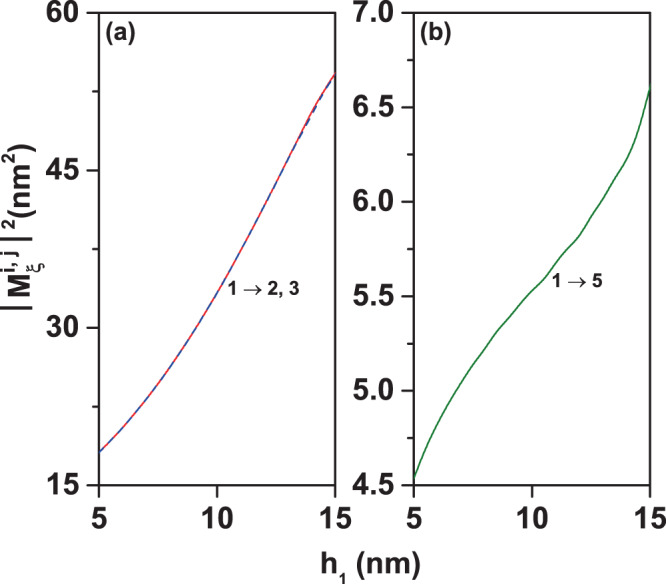
Transition matrix elements ${M}_{\xi }^{i,j}$ for confined electron states in a GaAs–Ga_0.7_Al_0.3_ As pyramidal core-shell quantum dot as functions of the innermost height (*h*_1_). Curves for the Ψ_1_ → Ψ_*j*_ (*j* = 2, ..., 13) transitions with *F* = 0, *B* = 0, *θ* = *π*/2, *h*_2_ = 20 nm, and *h*_3_ = 30 nm are shown. Two polarizations of the resonant incident radiation have been considered: circular polarized light in the *xy*-plane (**a**) and linearly *z*-polarized light (**b**).

Figure 6 shows the nonzero transition matrix elements (dipole moment divided by the electron charge, $|{M}_{\xi }^{i,j}{|}^{2}=|{\mu }_{\xi }^{i,j}/e{|}^{2}$) between the ground state (Ψ_*i*_, *i* = 1) and the first twelve excited states (Ψ_*j*_, *j* = 2, ..., 13) in a GaAs–Ga_0.7_Al_0.3_As PCSQD as functions of the structure’s innermost height (*h*_1_), for zero magnetic and electric fields, keeping fixed the other structure dimensions. In Fig. 6(a) the results are for circular polarization of the incoming light in the *xy*-plane while in Fig. 6(b) they correspond to linear polarization along the *z*-axis. From Fig. 6(a) we realize that nonzero off-diagonal matrix elements are present only for *j* = 2 and *j* = 3. The reason why, for example, ${M}_{x\pm i\,y}^{\mathrm{1,4}}=0$, comes from the fact that when calculating ${M}_{x}^{\mathrm{1,4}}$, Ψ_1_ is an even function with respect to *xz*-plane with *y* = 0 (Ψ_1_(*x*, −*y*, *z*) = Ψ_1_(*x*, *y*, *z*)), while with respect to the same plane, Ψ_4_ is an odd function (Ψ_4_(*x*, −*y*, *z*) = −Ψ_4_(*x*, *y*, *z*)) [with the global result ${M}_{x}^{\mathrm{1,4}}=0$; see first row of Fig. 4(a,b)]. When calculating ${M}_{y}^{\mathrm{1,4}}$, Ψ_1_ is an even function with respect to *yz*-plane with *x* = 0 (Ψ_1_(−*x*, *y*, *z*) = Ψ_1_(*x*, *y*, *z*)), whilst with respect to the same plane, Ψ_4_ is an odd function (Ψ_4_(−*x*, *y*, *z*) = −Ψ_4_(*x*, *y*, *z*)) [with the global result ${M}_{y}^{\mathrm{1,4}}=0$; see first row of Fig. 4(a,b)]. Because the wave functions Ψ_1_ and Ψ_5_ are even with respect to the *xz*-plane with *y* = 0 and the *yz*-plane with *x* = 0, it is obtained that ${M}_{x}^{\mathrm{1,5}}={M}_{y}^{\mathrm{1,5}}\mathrm{=0}$ and, consequently, ${M}_{x\pm i\,y}^{\mathrm{1,5}}\mathrm{=0}$ [see first row of Fig. 4(a,b)]. The symmetry arguments used to justify the null values of the matrix elements for the transitions Ψ_1_ → Ψ_4_ and Ψ_1_ → Ψ_5_ are the same arguments that can be applied to justify the non-zero values of the transitions Ψ_1_ → Ψ_2_ and Ψ_1_ → Ψ_3_. To discuss the reasons why the *z*-polarization induces or suppresses certain transitions, the symmetry properties of the wave functions are useful as well, taking into account, basically, the projections on the *xy*-plane; given that, because of the height of the pyramids, all the excited states under consideration have only one or two antinodes along the *z*-direction. In the case of a single node for the excited state, it appears displaced along the *z*-direction with respect to the ground state and when two nodes appear, clearly the corresponding wave function has opposite symmetry, along the *z*-direction, to that of the ground state. The increasing character of the matrix elements for circular polarization, shown in Fig. 6(a), results from the fact that, as the height of the innermost pyramid (*h*_1_) increases, the region where there is the highest probability of finding the electron moves away from the origin and thereby increases the overlap between the wave functions (see comparatively Ψ_1_ and Ψ_2_ in the first rows of Fig. 4(a,b)). A similar behavior occurs for the matrix element ${M}_{z}^{\mathrm{1,5}}$ in Fig. 6(b) (see comparatively Ψ_1_ and Ψ_5_ in the second rows of Fig. 4(a,b)).

**Figure 7:**
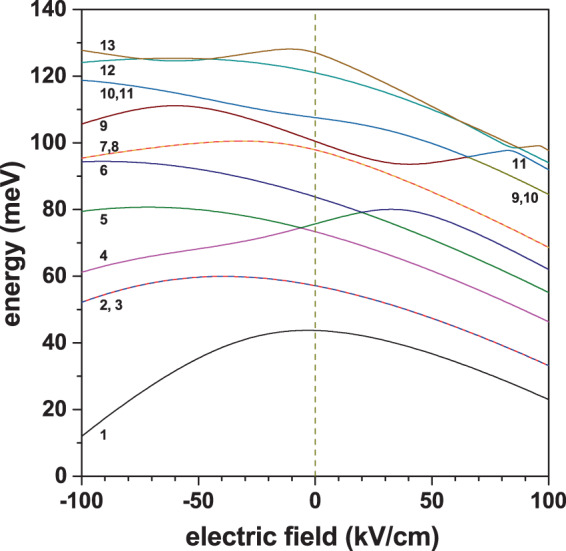
Energy of the lowest thirteen confined electron states in a GaAs–Ga_0.7_Al_0.3_ As pyramidal core-shell quantum dot as functions of the applied electric field (*F*). Results are depicted for *B* = 0, *θ* = *π*/2, *h*_1_ = 5 nm, *h*_2_ = 20 nm, and *h*_3_ = 30 nm.

In Fig. 7 we are presenting the energies of the first thirteen bounded states for an electron confined in a GaAs–Ga_0.7_Al_0.3_ As PCSQD as a function of the applied electric field strength. The results are for zero magnetic field and fixed dimensions of the structure. From the figure it is possible to observe some features that can be highlighted, such as: (*i*) throughout the whole range of the electric field intensity, the Ψ_2_ and Ψ_3_ states are doubly degenerate, the same as Ψ_7_ and Ψ_8_ states; (*ii*) for electric field strengths smaller than 65 kV/cm, the Ψ_10_ and Ψ_11_ states are degenerate and, for that specific value of the electric field they exchange symmetry with the Ψ_9_ state, giving rise to the doubly degenerated Ψ_9_ and Ψ_10_ states, for field values greater than 65 kV/cm; (*iii*) for *F* = −5.82 kV/cm an accidental degeneracy appears between Ψ_4_ and Ψ_5_ states, which is transferred to states Ψ_5_ and Ψ_6_ at *F* = 20.5 kV/cm; (*iv*) for *F* = 65.16 kV/cm a threefold degeneracy appears between Ψ_9_, Ψ_10_, and Ψ_11_ states; and, finally, (*v*) for *F* > 50 kV/cm, the behavior of the lowest eight states is linear and decreasing, thus showing a saturation effect with the electric field. It is important to note that the negatively oriented electric fields push the electronic states towards the top vertex of the pyramid, while the positive fields push them toward the bottom plane of the pyramid (see Fig. 1). Bearing in mind that at the top vertex of the pyramid the electronic states interact with four planes while at the bottom of the pyramid the interaction is with only one plane, this explains the reason why the energy curves are more sensitive to the electric field in the *F* > 0 regime. Examining, for example, the ground state, it is clearly noted that, for a finite value of the field, the energy curve is not symmetric with respect to *F* = 0. This is due, as already said, to the fact that the number of planes with which the particle interacts goes from four to one when the field changes from negative to positive values. The decreasing nature of this state with |*F*| is explained by the displacement towards lower energies of the bottom of the potential well related with the superimposition of the linear potential from the field with the confining potential of the structure.

**Figure 8:**
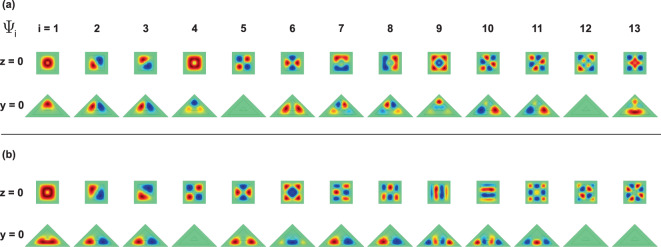
Pictorial view of the wave function projections (*z* = 0 and *y* = 0 planes) for the thirteen lowest confined electron states in a GaAs–Ga_0.7_Al_0.3_ As pyramidal core-shell quantum dot. The conditions for the calculation are: *F* = −100 kV/cm (**a**) and *F *= +100 kV/cm (**b**) with *B* = 0, *θ* = *π*/2, *h*_1_ = 5 nm, *h*_2_ = 20 nm, and *h*_3_ = 30 nm. The green color corresponds to a zero value of the wave function whereas the red and blue are associated to the positive and negative maxima, respectively.

Figure 8 shows the projections, on the *z* = 0 and *y* = 0 planes, of the first thirteen wave functions for an electron confined in a PCSQD with fixed values of the geometry, zero magnetic field, and considering two values of the applied electric field. In 8(a) the electric field pushes the carriers towards the apical region of the pyramid whereas in 8(b) these are pushed towards the pyramid base (see Figs. 1 and 2). Some of the main characteristics observed from the figure are the following: (*i*) For *F* = −100 kV/cm and *F* = +100 kV/cm, both the ground state and the first two excited states preserve their symmetries; something that is consistent with the absence of anticrossings between these states in Fig. 7, (*ii*) the states Ψ_2_ and Ψ_3_ are degenerate with *p*-like symmetry, (*iii*) when going from *F* = −100 kV/cm to *F* = +100 kV/cm the Ψ_5_ and Ψ_6_ states go to occupy the positions of the Ψ_4_ and Ψ_5_, respectively, and the Ψ_4_ state occupies the position of the Ψ_6_ one, consistently with the Fig. 7. It can be noticed that, at *F* = 20 kV/cm, Ψ_4_ exchanges symmetry with Ψ_6_, (*iv*) For *F* = −100 kV/cm and *F* = +100 kV/cm, the numerical method used introduces a phase of ±*π*/4 for Ψ_2_ and Ψ_3_ states in the *z* = 0 plane. This phase is also present in the Ψ_10_ and Ψ_11_ states at *F* = −100 kV/cm and Ψ_12_ and Ψ_13_ states at *F* = +100 kV/cm, (*v*) when comparing Ψ_1_, Ψ_2_, and Ψ_3_, in Fig. 8(a,b), it is clearly seen how, in the first case, the states are displaced towards the pyramid apex while in the second case they are directed towards the pyramid basal plane, (*vi*) the presence of only one antinode in the *z*-direction of the Ψ_1_, Ψ_2_, and Ψ_3_ states -given the odd symmetry of Ψ_2_ and Ψ_3_ with respect to a ±*π*/4 rotated plane =, ensures that over the entire range of applied electrical fields, there is a non-zero value of the matrix elements for *xy*-circularly polarized incident radiation, as will be seen below, and (*vii*) in general, for all excited states, the energy is higher at *F* = −100 kV/cm with respect to *F* = +100 kV/cm, due to the greater interaction with the lateral planes at the pyramid apex. Note that the electric field implies a remarkable change of the wave function characteristics. It can be affirmed that for negative electric field strengths, the electronic probability is distributed in a 3D-region whereas for sufficiently high positive electric fields, the spatial distribution of the states primarily locates nearby the pyramid basal plane. With the electric field, the system evolves from a three-dimensional quantum dot (for negative fields) to a two-dimensional quantum dot (for positive fields).

**Figure 9:**
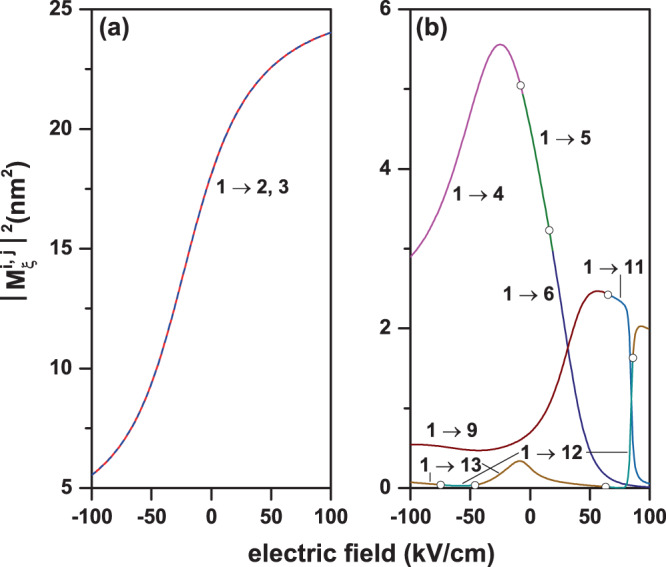
Transition matrix elements ${M}_{\xi }^{i,j}$ for confined electron states in a GaAs–Ga_0.7_Al_0.3_ As pyramidal core-shell quantum dot as functions of the applied electric field (*F*). Calculations are for the Ψ_1_ → Ψ_*j*_ (*j* = 2, ..., 13) transitions with *B* = 0, *θ* = *π*/2, *h*_1_ = 5 nm, *h*_2_ = 20 nm, and *h*_3_ = 30 nm. Two polarizations of the resonant incident radiation have been considered: circular polarized light in the *xy*-plane (**a**) and linearly *z*-polarized light (**b**).

When going from negative to positive electric field values, the superposition between Ψ_1_ and Ψ_2_ (Ψ_1_ and Ψ_3_) states increases along with the increase in the spatial extent of the states. This justifies the ever increasing character of transition matrix elements ${M}_{x\pm i\,y}^{\mathrm{1,2}}$ and ${M}_{x\pm i\,y}^{\mathrm{1,3}}$ in Fig. 9(a). For $F\cong 100$ kV/cm, a saturation effect of these matrix elements is observed due to the lateral potential barriers influence on the WFs. For *F* = 66 kV/cm, it is observed that the transitions Ψ_1_ → Ψ_10_, Ψ_11_ are transformed into Ψ_1_ → Ψ_9_, Ψ_10_, which is in agreement with the crossing observed in Fig. 7 for such electric field value, at *E* = 96 meV. For circular polarization (see Fig. 9(a)) and *F* = −14 kV/cm only the Ψ_1_ → Ψ_2_, Ψ_3_ transitions are present, the other transitions are suppressed, this despite the fact that the symmetries in each *z*-plane are preserved with the electric field, but they change as the plane moves in that direction giving rise to contributions that cancel each other out. In Fig. 9(b), for *z*-polarized incident light, it is observed, for example, that the Ψ_1_ → Ψ_4_ transition is transformed into the Ψ_1_ → Ψ_5_ transition, and this finally becomes the Ψ_1_ → Ψ_6_ transition. This behavior is in agreement with the observed crossings between the Ψ_4_, Ψ_5_, and Ψ_6_ states in Fig. 7 for *F* = −6.4 kV/cm and *F* = 19.5 kV/cm. This situation is also evident for other permitted transitions either with circular or linear incident polarized light, as shown in the two panels of Fig. 9. The increase of ${M}_{z}^{\mathrm{1,4}}$ in the negative range of applied electrical fields is due to the fact that initially, for *F* = −100 kV/cm, the maximum probability of both states is located at the apex of the pyramid; as *F* grows towards zero, the Ψ_1_ state extends over the entire central pyramid while Ψ_4_ remains almost static at the apex of the pyramid (note that the Ψ_4_ state has two antinodes in the *z*-direction, which guarantees the non-null value of the matrix element). The curve reaches a maximum at *F* = −23 kV/cm where precisely the ground state has its maximum spatial distribution. For *F* > 0, where the character of the transition is Ψ_1_ → Ψ_5_ and then Ψ_1_ → Ψ_6_, the decreasing behavior of ${M}_{z}^{\mathrm{1,5}}$ and ${M}_{z}^{\mathrm{1,6}}$ is due to the fact that the ground state is compressed towards the base of the pyramid and the excited state in question undergoes a progressive displacement towards the base of the pyramid as the field grows. Similar analysis, based on the distributions of the wave functions and their symmetries, explain the behavior of the other matrix elements.

**Figure 10:**
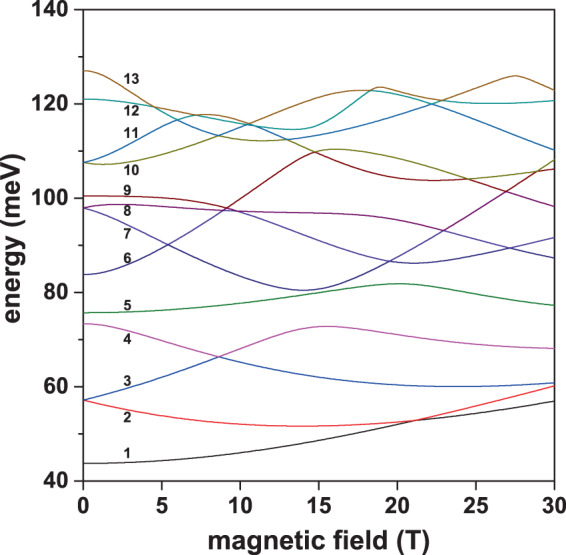
Energy of the lowest thirteen confined electron states in a GaAs–Ga_0.7_Al_0.3_ As pyramidal core-shell quantum dot as a function of the applied magnetic field (*B*). Results correspond to *F* = 0, *θ* = *π*/2, *h*_1_ = 5 nm, *h*_2_ = 20 nm, and *h*_3_ = 30 nm.

**Figure 11:**
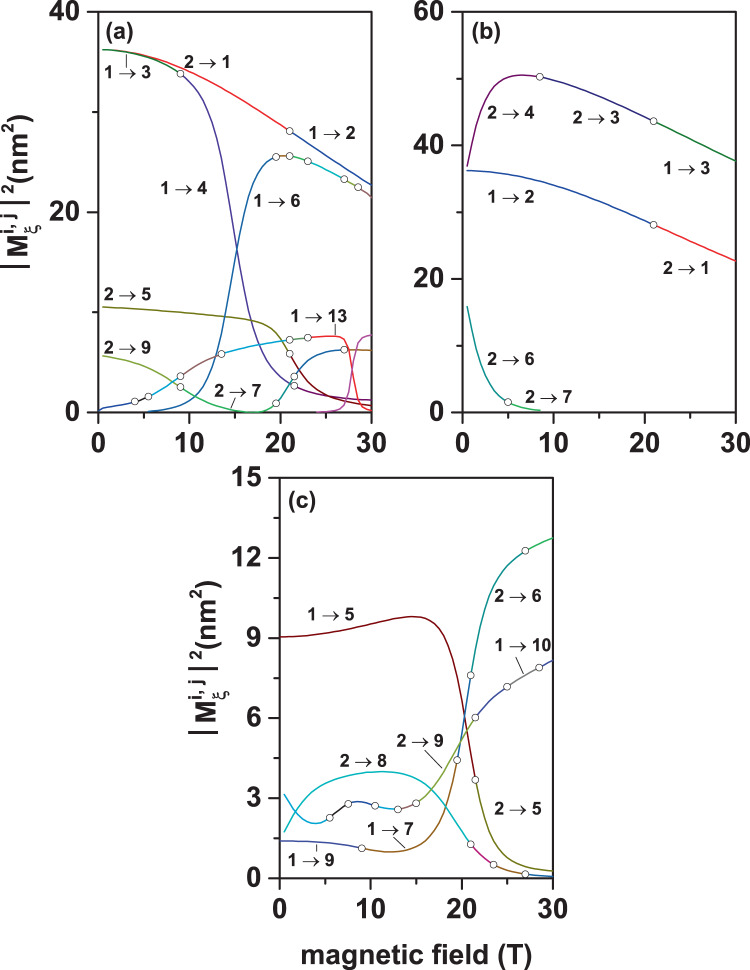
Transition matrix elements ${M}_{\xi }^{i,j}$ for confined electron states in a GaAs–Ga_0.7_Al_0.3_ As pyramidal core-shell quantum dot as functions of the applied magnetic field (*B*). Curves represent the Ψ_1_ → Ψ_*j*_ (*j* = 2, ..., 13) transitions with *F* = 0, *θ* = *π*/2, *h*_1_ = 5 nm, *h*_2_ = 20 nm, and *h*_3_ = 30 nm. Three different polarizations of the resonant incident radiation have been considered: left-handed circular polarized light in the *xy*-plane (**a**), right-handed circular polarized light in the *xy*-plane (**b**), and linearly *z*-polarized light (**c**).

**Figure 12:**
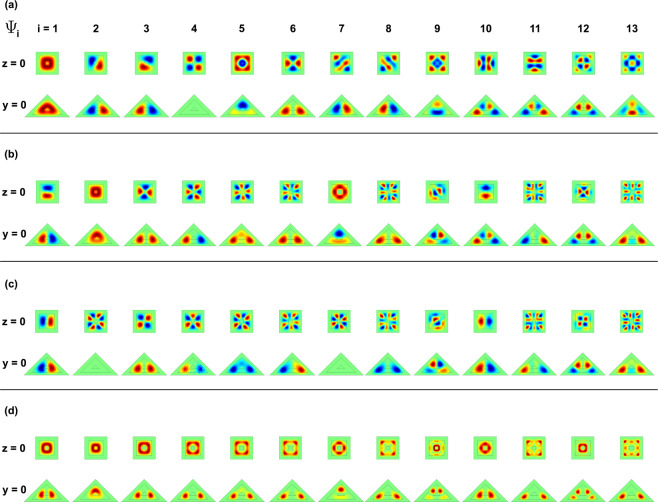
Pictorial view of the wave function projections (*z* = 0 and *y* = 0 planes) for the thirteen lowest confined electron states in a GaAs–Ga_0.7_Al_0.3_ As pyramidal core-shell quantum dot. Calculations are for *B* = 0 and *B* = 30 T with *F* = 0, *θ* = *π*/2, *h*_1_ = 5 nm, *h*_2_ = 20 nm, and *h*_3_ = 30 nm. The results are as follows: Ψ_*i*_ for *B* = 0 (**a**), $\Re ({\Psi }_{i})$ for *B* = 30 T (**b**), ℑ$({\Psi }_{i})$ for *B* = 30 T (**c**), and Ψ_*i*_-probability density for *B* = 30 T (**d**). The green color corresponds to a zero value whereas the red and blue are associated to the positive and negative maxima, respectively.

In Figs. 10–12, we present the study of the applied magnetic field effects on the electronic states in a GaAs–Ga_0.7_Al_0.3_ As PCSQD. The magnetic field is applied in the *z*-direction, which coincides with the symmetry axis of the heterostructure. This guarantees that the symmetry of the states in the different planes where *z* = *const*. is preserved. In Fig. 10, the energies for the first thirteen confined states are reported, in Fig. 11 the figures correspond to results proportional to the dipole matrix elements considering circular and linear polarization for the incident radiation. Finally, Fig. 12 contains the projections of the wave functions on the *z* = 0 and *y* = 0 planes.

In Fig. 10 it is observed that the first relevant effect of the magnetic field is the breakdown of degeneracy for all reported states. Besides, much of the corresponding off-diagonal dipole moment matrix elements appear to be different to zero, as functions of *B*, as can be readily noticed from Fig. 11, and will be discussed below.

To interpret this situation, we resort to the wave functions and probability densities depicted in Fig. 12. Note that in Fig. 12(a), at zero magnetic fields, the states Ψ_2_ and Ψ_3_ (which correspond to real wave functions) have the same configuration of nodes and antinodes and are characterized by being rotated with respect to each other at an angle of 90°, taking the symmetry axis as the axis of rotation. This, as previously analyzed, explains the degeneration of the states. The same situation is valid for the Ψ_7_ and Ψ_8_ states of Fig. 12(a).

When the magnetic field is turned on (*B* = 30 T), one may observe that the wave functions become complex, with real and imaginary components, as represented in Fig. 12(b,c). Analyzing the Ψ_1_ and Ψ_4_ states (which at zero magnetic field correspond to the Ψ_2_ and Ψ_3_ states), it is observed that both the real and imaginary part of both states are displaced towards the base of the pyramid. It is also appreciated that while for Ψ_1_ the real part of the wave function is always positive, in the case of Ψ_4_ there are three regions of maximum positive and three regions of maximum negative contributions. In the case of the imaginary parts, the projections in the *z* = 0 plane show a positive maximum and a negative maximum for the Ψ_1_ state while for Ψ_4_ there are three positive and three negative maxima. The combination of the real and imaginary parts, which corresponds to the probability density (as shown in Fig. 12(d)), leads to the fact that the Ψ_1_ state (of lower energy) is located in the region near the axis of the pyramid with a wide volumetric distribution of the probability density. In the same manner, for the Ψ_4_ state (of larger energy), the electron tends to concentrate in a thin layer near the base of the pyramid, with well-defined maxima near the vertices of the square cross section. A similar situation is exhibited by the Ψ_7_ and Ψ_8_ states which, when the magnetic field is turned on until *B* = 30 T, evolve to become the Ψ_10_ and Ψ_6_ states, respectively.

Then, a second point to highlight in Fig. 10 is the presence of ground state oscillations as the magnetic field increases. Note from Fig. 11 that, for *B* = 30 T, the ground state (Ψ_1_) has a real part whose symmetry coincides with that of the ground state (Ψ_1_) at *B* = 0 and that the imaginary part of Ψ_1_ at *B* = 30 T has the same *p*-like symmetry of Ψ_2_ at *B* = 0. This explains the change in symmetry presented by the ground state at *B* = 21.2 T. As a third aspect, note also the presence of anticrossings between states which are induced by the magnetic field effects. Near *B* = 15 T, an anticrossing appears between the states that have been labeled as Ψ_3_ and Ψ_7_ at zero magnetic field. At *B* = 21 T there is another anticrossing, this time between Ψ_5_ and Ψ_9_. Finally, Fig. 10 shows multiple accidental degeneracies. For example, the ground state presents accidental degeneracy at *B* = 21 T. What is most relevant to our investigation is that all these crossings or anticrossings between states are reflected in changes in the symmetry of the wave functions and, consequently, in changes in the selection rules for optical transitions between states.

This can be clearly seen in Fig. 11, where the squared absolute expected values of the dipole matrix elements with *ξ* = *x* ± *iy* (for circular polarization) and *ξ* = *z* (for linear polarization) are presented for transitions between the ground state and the first twelve excited states and between the first excited state and the next eleven excited states. Unlike the cases discussed in Figs. 6 and 9, here it has been necessary to include transitions from the first excited state given the crossing between Ψ_1_ and Ψ_2_ at *B* = 21 T. The complex character, with real and imaginary parts, of the wave functions, explains the absolutely different response presented by the system to light with right and left circular polarization, as seen from Fig. 11(a,b). Note that each line in the three panels of this figure is composed of transitions between multiple different states. Each open symbol indicates the change between states involved in transitions. Each open symbol appears for magnetic field and energy values that correspond to the crossings between states in Fig. 10. Looking at Fig. 11(c) for example, one may observe that, under field conditions, essentially four well-defined transitions appear while at *B* = 30 T only two transitions will be noticeable. This evidences a remarkable change in selection rules as the magnetic field grows. In the case of Fig. 11(a), at *B* = 0 there are four well-defined transitions that evolve into four others, but between different energy states.

### The optical coefficients

**Figure 13:**
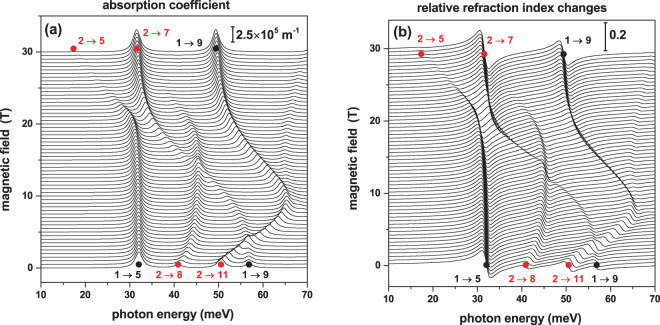
Calculated light absorption (**a**) and relative refraction index change (**b**) coefficients associated to transitions between single electron states in a GaAs–Ga_0.7_Al_0.3_ As pyramidal core-shell quantum dot, depicted as functions of the *z*-polarized incident photon energy, for several values of the applied magnetic field between zero and 30 T. The setup considers *F* = 0, *θ* = *π*/2, *h*_1_ = 5 nm, *h*_2_ = 20 nm, and *h*_3_ = 30 nm. The vertical bar in each panel shows the corresponding scale of the depicted optical property. Full symbols indicate the energy position of the nonzero corresponding matrix elements at zero (black) and 30 T (red), reported in Fig. 11(c).

In Fig. 13, the light absorption (a) and relative refraction index change (b) coefficients appear plotted as functions of the *z*-polarized incident photon energy and the applied magnetic field. The calculations considered the situation with zero applied electric field and kept constant the geometry and dimensions of the structure. Note that at *B* = 0, the peak of greater amplitude, both in the case of the absorption coefficient and the relative refractive index changes, is given for the 1 → 5 transition. This is consistent with the content of Fig. 11(c) where the most significant value of ${M}_{z}^{i,j}$ at *B* = 0 is, precisely, given for the 1 → 5 transition. Besides, For *B* = 30 T, in Fig. 13(a) two peaks with approximately the same intensity are observed. This is despite the fact that in Fig. 11(c) the 1 → 9 transition has a matrix element smaller than that of the 2 → 7 transition, whose energy is lower than the one corresponding to the 1 → 9 transition.

Taking into account that the magnitude of *α*(*ω*) is proportional to the product ${E}_{fi}\,|{M}_{z}^{fi}{|}^{2}$, at *B* = 30 T the 2 → 7 and 1 → 9 transitions are proportional to 398.7 nm^2^ meV and 401 nm^2^ meV, respectively. Additionally, it can be seen from Fig. 13(a) that the 1 → 5 and 1 → 9 transitions, at *B* = 0 evolve to the 2 → 5 and 2 → 7 transitions at *B* = 30 T, which is consistent with the anti-crossing taking place near *B* = 20 T with *E*_65_ = 80 meV between the Ψ_5_ and Ψ_6_ states.

On the other hand, when observing Fig. 13(b), one may see that the relative refractive index changes peak amplitudes exactly follow the behavior presented by those corresponding to the optical absorption coefficient in Fig. 13(a). This comes from the fact that for a particular *i* → *j* transition, the coefficient of relative refraction index change is an odd function with respect to the transition energy, *E*_*fi*_ = *E*_*f*_  − *E*_*i*_, the same at which the absorption coefficient shows the resonant peak structure. Also, it is clear that the Δ*n*/*n*_*r*_ coefficient has a maximum and a minimum localized at *E*_*p*_ = *E*_*fi*_ − *ℏ*Γ and *E*_*p*_ = *E*_*fi*_ + *ℏ*Γ, respectively. Additionally, taking into account that the magnitude of the two resonant peaks of Δ*n*/*n*_*r*_ are proportional to $|{M}_{z}^{i,j}{|}^{2}$, the reason why the peaks of the 2 → 7 transition are significantly greater than the peaks of 1 → 9 at *B* = 30 T is explained.

## Conclusions

We have performed the investigation of electron states in core-shell pyramidal quantum dots considering the effect of externally applied electric and magnetic fields to the structure. The results of the calculation included modifications of the system size and geometry as well. Accordingly, we present a detailed discussion about the properties of energies and wave functions under different configurations, making emphasis in those related with the symmetry of states and how they are modified by the application of the external probes, showing both crossings and anticrossings in their evolution as functions of the field strengths. Regarding this, the study finds that a number of inter-state transitions can become forbidden, and the presence of an external probe, with its associated degeneracy breaking, activates some of them.

The information about the electronic structure allows to evaluate the coefficients of light absorption and relative refractive index change associated to allowed transitions between the lowest confined states. We comment on the features of the peaks and amplitudes of these optical responses, as functions of the incident light energy for the particular presence of an applied magnetic field.

## Data Availability

All the files with tables, figures, and codes are available. The corresponding author will provide all the files in case they are requested.
